# ‘In research, publishing and practice, the individual always matters’

**DOI:** 10.1111/hex.13004

**Published:** 2019-11-28

**Authors:** Carolyn A. Chew‐Graham

**Affiliations:** ^1^ School of Primary, Community and Social Care Faculty of Medicine and Health Sciences Keele University Keele Staffordshire UK

The importance of exploring the individual experience in qualitative studies is widely acknowledged. A recent Editorial in The Lancet Psychiatry states ‘In research, publishing, and practice, the individual always matters’.^1^

There are a number of excellent qualitative studies in this edition of HEX, exploring the perspectives of the individual. Im et al, report a study exploring the challenges of living with heart failure from the perspectives of patients, with some dyadic interviews including family caregivers. The authors suggest that whilst patients and caregivers may have knowledge of heart failure management, there may be gaps in patients' and caregivers’ understanding of the consequences of illness. The authors suggest that clinicians should be aware of what older adults and caregivers understand about the consequences of their illness to ensure that planned management aligns with an individual's preferences and needs, as they age and advance in their illness towards the end‐of‐life. The authors suggest that their findings align with the Reconceptualized Uncertainty in Illness Theory.^2^, ^3^ Since uncertainty characterizes many long‐term conditions, there is a need to reconceptualize the theory of uncertainty to include the experience of living with continual uncertainty.

Currie et al interviewed fifteen parents of children with neurodevelopmental disorders. The authors describe three insights from their analysis: parents experience a sense of disconnect and ‘silencing’ as little is known or understood by health‐care providers about the experience of caring for children at home; parents make strong efforts to be heard and acquire services within health and social systems describing themselves as ‘fighters’, ‘saviours’ and ‘navigators’; and parents sacrifice themselves to the caregiving role and become therapists and caregivers to their children at the cost of losing themselves as parents. As with the Im study, Currie et al suggest that their study has lessons for health‐ and social care professionals—the need to understand parent's experiences and challenges, in order to plan acceptable and appropriate management.

Johnsson et al report a study exploring encounters between patients, relatives and nurses using an ethnographic approach, emphasizing the need for nurses to understand the patients’ and relatives’ stories, in order to strengthen the patient's position in the health‐care setting. The authors provide a useful model to illustrate their findings.

A focus group study, described by Årestedt et al, with 42 key informants including patients, staff and managers with the experience of dialysis care. The concept of patient participation was reflected upon by staff and patients, with staff feeling that undergoing dialysis was evidence of participation, whilst patients viewed ‘participation’ in broader terms, starting from the sharing of information and being supported to make a decision about dialysis.

Other articles published in this issue of HEX involve the use of peer researchers. Peer research is defined by the McPin Foundation^4^ as, ‘research that is steered and conducted by people with lived experience of the issue being studied’. McPin literature describes both benefits and potential challenges to the use peer research, drawing from published research and the direct experience of the authors.

A study by Eades et al used a mixed‐methods cross‐sectional design to explore non‐attendance at diabetes appointments in Scotland. People with diabetes (peer researchers) were involved in the conception and design of the study and were trained to carry out semi‐structured telephone interviews in the first phase of the study. The findings of these interviews informed the content of a questionnaire in the second phase of the study. The paper includes an outline of the training, which was offered to the peer researchers. Analysis of the data led to a description of perceived impact of diabetes, practical barriers of attending appointments and the perceived value of appointments. Eades at al underpinned their analysis with the Theory of Planned Behaviour (TPB)^5^ and the Self‐Regulation Model of Illness Behaviour (SRM).^6^

The importance of patient and public involvement and engagement in research—in the design and conduct of a study, in the interpretation of findings and in the dissemination of findings—is increasingly recognized.^7^ Patient and public involvement (PPI) in research (also known as service user/lay involvement) refers to an active partnership between patients and/or members of the public and researchers. In this edition of HEX, Lee et al report a community PPIE in which the knowledge, barriers and promoters for optimal vitamin D status in a Somali population were explored. A focus group, facilitated by two researchers and a PPIE lead, described the barriers to adequate sunlight exposure and access to health care; the researchers emphasize that the public voice can influence public health interventions and identify how to target health messages.

The importance of managing expectations of people contributing to PPIE activity is highlighted by Poland et al who describe the complexity of public involvement and suggest that whilst involvement can empower the individual, it cannot be assumed to be a simple or universal ‘panacea’ for increasing the relevance and accessibility of research to the public.

Finally, there is a move towards co‐production of research: INVOLVE describes co‐producing a research project as ‘an approach in which researchers, practitioners and the public work together, sharing power and responsibility from the start to the end of the project, including the generation of knowledge’^8^; INVOLVE outlines key principles including *sharing of power*, *reciprocity* and *maintaining and building relationships*; and key features including *establishing ground rules, ongoing dialogue, joint ownership of key decisions* and *evaluating impact of co‐produced research.*

In this edition of HEX, Suutari et al report findings from their co‐produced research utilizing a ‘Learning café’ group education programme^9^ which included patients with atrial fibrillation. The programme was reported to improve ‘sense of security’ in patients, and anticipating a shift from emergency to planned care and increase work satisfaction in health‐care professionals.

Finally, I would draw readers’ attention to a recent paper by Locock and Boaz,^10^ which asserts that different approaches to involvement such as patient and public involvement and engagement (PPIE), qualitative research, participatory research, co‐design and co‐production sit alongside each other, but boundaries can be unclear. There has also been a subtle shift in the discourse, with the language of co‐design and co‐production used more widely in debates about involvement. Locock and Boaz argue that encouraging ‘non‐scientific actors’ to participate in the knowledge production process can have a transformative impact on knowledge, enhancing its relevance, reliability and scope for impact. All these approaches—PPIE, qualitative research, user testing and co‐design—may share similar values but represent competing understandings and beliefs of how to get there. Rycroft‐Malone et al^11^ suggest that the approach taken should be driven by methodological and practical considerations, and a concern for ‘authentic collaboration’ which emphasizes ‘the importance of engaging and integrating the multiple perspectives of stakeholders that can shape the understanding and process of knowledge generation and use’.

The key message: HEX welcomes submissions reporting the experiences of the individual, with studies utilizing qualitative research methods, PPIE, co‐design and co‐production.
